# Immune environment modulation in pneumonia patients caused by coronavirus: SARS-CoV, MERS-CoV and SARS-CoV-2

**DOI:** 10.18632/aging.103101

**Published:** 2020-05-02

**Authors:** Zhixian Yao, Zhong Zheng, Ke Wu, Zheng Junhua

**Affiliations:** 1 Shanghai General Hospital, Shanghai Jiao Tong University, School of Medicine, Shanghai, China

**Keywords:** COVID-19, SARS-Cov-2, cytokine storm

## Abstract

Currently, we are on a global pandemic of Coronavirus disease-2019 (COVID-19) which causes fever, dry cough, fatigue and acute respiratory distress syndrome (ARDS) that may ultimately lead to the death of the infected. Current researches on COVID-19 continue to highlight the necessity for further understanding the virus-host synergies. In this study, we have highlighted the key cytokines induced by coronavirus infections. We have demonstrated that genes coding interleukins (Il-1α, Il-1β, Il-6, Il-10), chemokine (Ccl2, Ccl3, Ccl5, Ccl10), and interferon (Ifn-α2, Ifn-β1, Ifn2) upsurge significantly which in line with the elevated infiltration of T cells, NK cells and monocytes in SARS-Cov treated group at 24 hours. Also, interleukins (IL-6, IL-23α, IL-10, IL-7, IL-1α, IL-1β) and interferon (IFN-α2, IFN2, IFN-γ) have increased dramatically in MERS-Cov at 24 hours. A similar cytokine profile showed the cytokine storm served a critical role in the infection process. Subsequent investigation of 463 patients with COVID-19 disease revealed the decreased amount of total lymphocytes, CD3+, CD4+, and CD8+ T lymphocytes in the severe type patients which indicated COVID-19 can impose hard blows on human lymphocyte resulting in lethal pneumonia. Thus, taking control of changes in immune factors could be critical in the treatment of COVID-19.

## INTRODUCTION

The family of coronaviruses (CoV) are enveloped RNA viruses which can be highly pathogenic to human beings [1]. Before long, the epidemics of the two highly infectious coronaviruses, severe acute respiratory syndrome coronavirus (SARS-CoV) [2] and Middle East respiratory syndrome coronavirus (MERS-CoV) [3] had resulted disastrous effects to human beings globally. The outbreak of Severe Acute Respiratory Syndrome Coronavirus 2 (SARS-CoV-2) and Coronavirus disease-2019 (COVID-19) originated from Wuhan, China in the end of 2019 has caused thousands of deaths [4]. Phylogenetic analysis of SARS-CoV-2 indicated that it is closely related to SARS-CoV (~79%) and a little more distant to MERS-CoV(~50%) [5]. The pathological changes of COVID-19 dead puncture suggest that its pathological characteristics are very similar to SARS-CoV and MERS-CoV-induced viral pneumonia [6]. Thus, it is critical to identify common patterns between these lethal pathogens and immune response.

Coronavirus has specific immune response and immune escape characteristics, and then causes severe pathogenic mechanisms through inflammation, which leaded to severe pneumonia, pulmonary oedema, ARDS, or multiple organ failure and even death [7]. Cytokine storm, also known as cytokine cascade, or hypercytokinemia, is caused by infection, drugs or autoimmune diseases of the body’s excessive immunity response [8]. Pioneering investigations have confirmed that increased volumes of pro-inflammatory cytokines in serum (e.g., IL-1B, IL-6, IL-12, IFN-γ) correlated with pulmonary inflammation and severe lung impairment in SARS patients [9]. MERS-CoV infection was also described to provoke increased concentrations of cytokines (IL-15,IL-17, TNF-α, and IFN-γ) [10]. It is reported that victims infected with SARS-CoV-2 also demonstrate high amounts of IL-1B, IFN-γ, IP10, and MCP1, which may attribute to activated Th1 (T helper) cell responses [11]. Although these virus invaded human bodies through various proteins(SARS-CoV: angiotensin-converting enzyme 2, Angiotensin-Converting Enzyme 2 (ACE-2), MERS-CoV: Dipeptidyl Peptidase-4 (DDP-4), SARS-CoV-2: ACE-2 possibly), the similar cytokine cascade from immune response which caused severe damage has been widely covered [12].

Hence, identifying the key cytokines induced by coronavirus infection and the cells involved in the regulation of cytokine storms, blocking their signal transduction, will greatly reduce the inflammatory response and damage to the lung tissue and multiple organs of patients.

## RESULTS

### Invasion process and immune response of SARS-CoV, MERS-CoV and SARS-CoV-2

**Figure 1:**
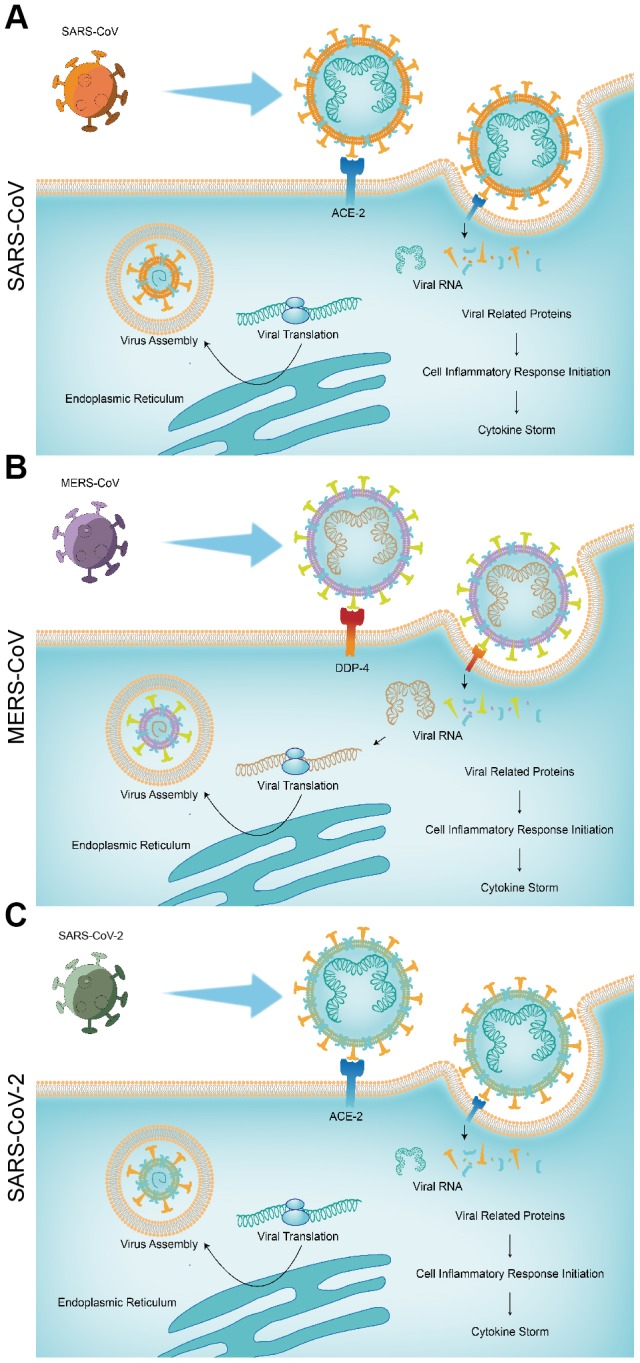
**The pathogenic mechanisms of the three pneumonias.** (**A**) SARS-CoV; (**B**) MERS-CoV; (**C**) SARS-CoV-2.

SARS-CoV-2 shows 88% identity to the sequence of SARS-like coronaviruses and about 50% to the sequence of MERS-CoV. Due to the similar structure, their pathogenesis is similar. SARS-CoV-2, just like SARS-CoV, requires the ACE-2. MERS-CoV enters target cells not via ACE-2, but via binding to DPP-4. Both ACE-2 and DPP-4 are expressed in several human tissues. While the virus enters the cells, antigen presentation subsequently stimulates the body’s humoral and cellular immunity, which are mediated by virus-specific immune cells. Immune response causes a lot of symptoms and the main death cause of coronavirus is cytokine storm, which is the deadly uncontrolled systemic inflammatory response. COVID-19 induced strong immune response is resulting from the release of large amounts of pro-inflammatory cytokines and chemokines, which are similar to the symptoms of SARS-CoV and MERS-CoV infections. Hence, although the pathogenesis of COVID-19 is poorly understood, the similar mechanisms of SARS-CoV and MERS-CoV still can give us a lot of information on the pathogenesis of SARS-CoV-2 infection to facilitate our recognition of COVID-19 (Figure 1).

### SARS-CoV-induced immune responses

**Figure 2:**
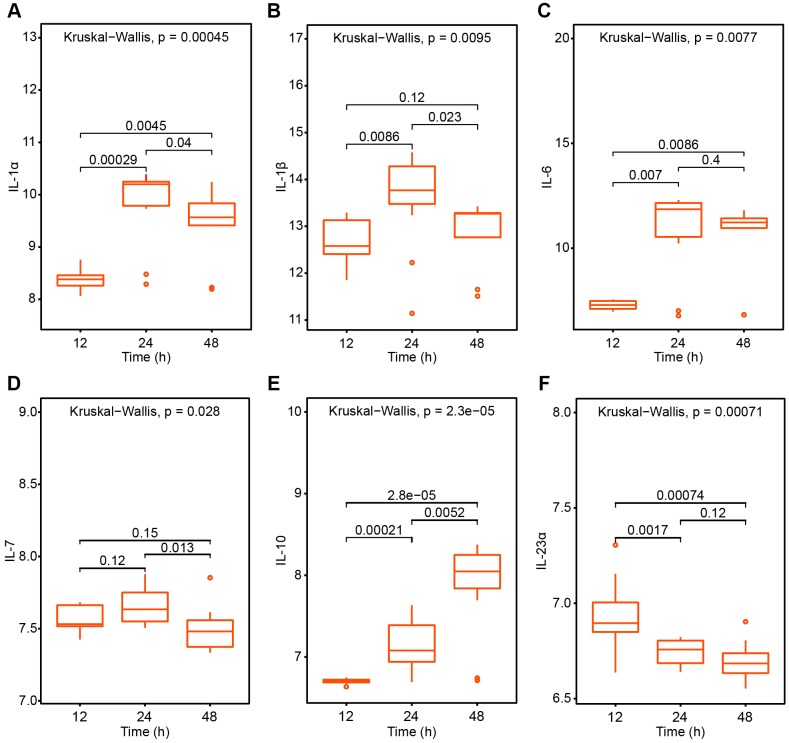
**The pneumonia related interleukin cytokines variation trend after SARS-CoV treatment 12h, 24h and 48h respectively.** (**A**) IL-1α; (**B**) IL-1β; (**C**) IL-6; (**D**) IL-7; (**E**) IL-10; (**F**) IL-23α.

To explore SARS-CoV induced immune responses, infected mice group was analyzed. Lungs from mice were harvested at 12, 24, and 48 hours post-infection and at least 3 biological replicates were collected. As pneumonia in the elderly is more susceptible to infection and the symptoms are heavier, the changes in inflammatory factors at 12, 24, and 48 hours after the infection of the SARS virus in elderly rats were analyzed, and multiple factors were found to occur. IL-1α, IL-1 β, IL-6 and IL-10 presented a significant higher level and was more obvious at 24 hours while the level of IL-7 showed moderate fluctuation and IL-23α a decreased trend (Figure 2). The results showed that SARS-CoV infection induced a cytokine storm.

**Figure 3:**
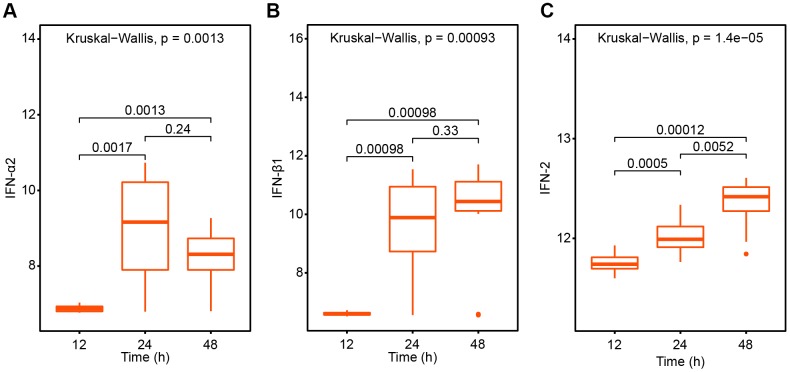
**The interferon variation trend after SARS-CoV treatment 12h, 24h and 48h respectively.** (**A**) IFN-α2; (**B**) IFN-β1; (**C**) IFN-2.

**Figure 4:**
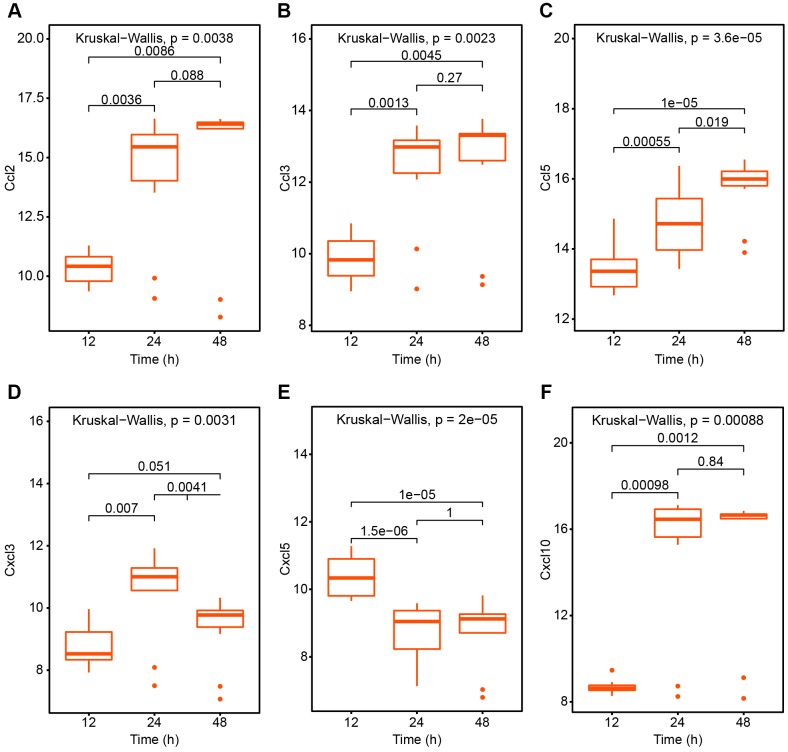
**The variation trend of chemokines after SARS-CoV treatment 12h, 24h and 48h respectively.** (**A**) Ccl2; (**B**) Ccl3; (**C**) Ccl5; (**D**) Cxcl3; (**E**) Cxcl5; (**F**) Cxcl10.

As for interferon system which protects mammals against virus infections, we analyzed the changes of interferon at 12h, 24h and 48h after infection with SARS virus in elderly rats. We found IFN-α2, IFN-β1 and IFN2 all demonstrated higher expression volumes especially in 24h (Figure 3) which suggest the onset such as plasmacytoid dendritic cells (pDCs) and proinflammatory monocytes. In terms of changes in chemokines which synergistically induce a proinflammatory recruitment, the level of CCL2, CCL3, CCL5 and CCL10 are all drastically elevated in 24h and remained high level in 48h. In the meantime, CXCL3 expression increased in 24h but decreased in 48h. And CXCL5 expression showed a decreased trend in 24h and 48h compared to 12h (Figure 4). Taken together, these rising molecules reflected anti-viral response from the host in the early phase.

### MERS-CoV-induced immune responses

**Figure 5:**
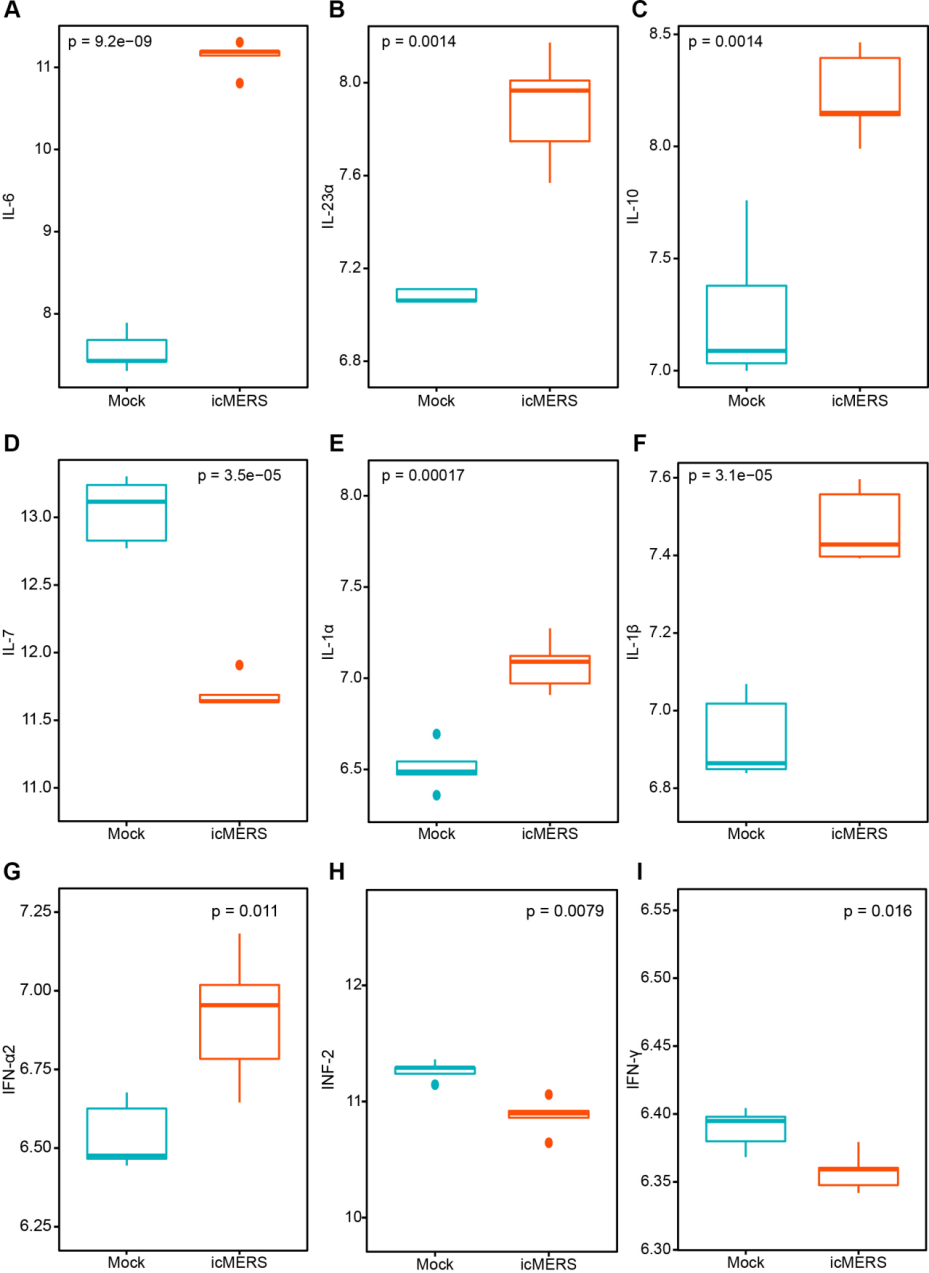
**The interleukin cytokines and interferon variation trend after MERS-CoV treatment in 24 hours.** (**A**) IL-6; (**B**) IL-23α; (**C**) IL-10; (**D**) IL-7; (**E**) IL-1α; (**F**) IL-1β; (**G**) IFN-α2; (**H**) IFN-2; (**I**) IFN-γ. (Mock: Control group; icMERS: MERS-CoV treated group).

In order to explore the common pattern of immune response after coronavirus contagion, we analyzed the situation in MERS-CoV infected human microvascular endothelial cells. So we analyzed the expression genes of interleukins and interferons after 24h. And we found interleukins (IL-6, IL-23α, IL-10, IL-7, IL-1α, IL-1β) and interferons (IFN-α2, IFN2, IFN-γ) have increased dramatically (Figure 5) which indicated an elevated anti-virus immune response.

### Differences in immune responses in young and aged mice

**Figure 6:**
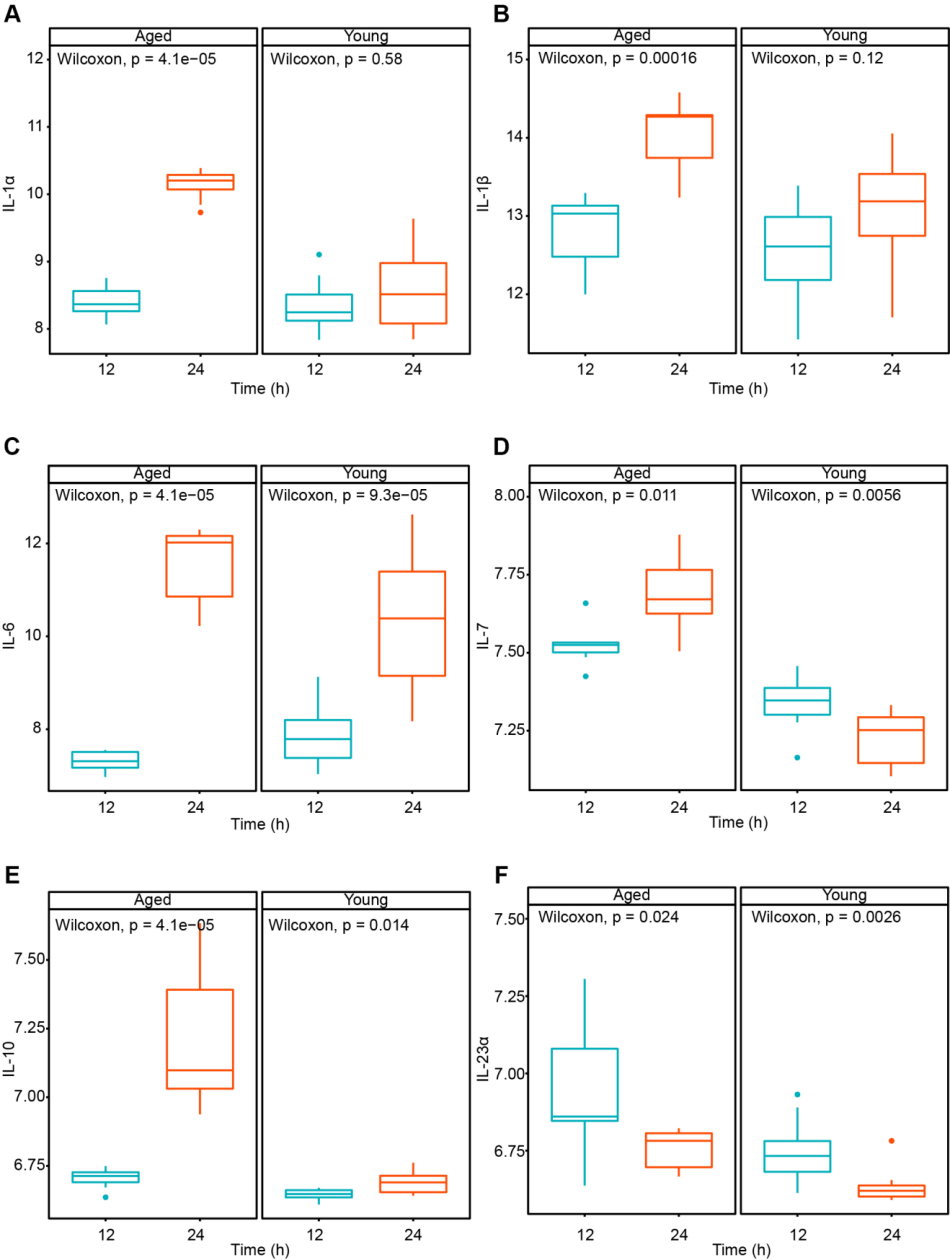
**Cytokine variation in young and aged mice after MERS-CoV treated for 12 and 24 hours.** (**A**) IL-1α; (**B**) IL-1β; (**C**) IL-6; (**D**) IL-7; (**E**) IL-10; (**F**) IL-23α.

**Figure 7:**
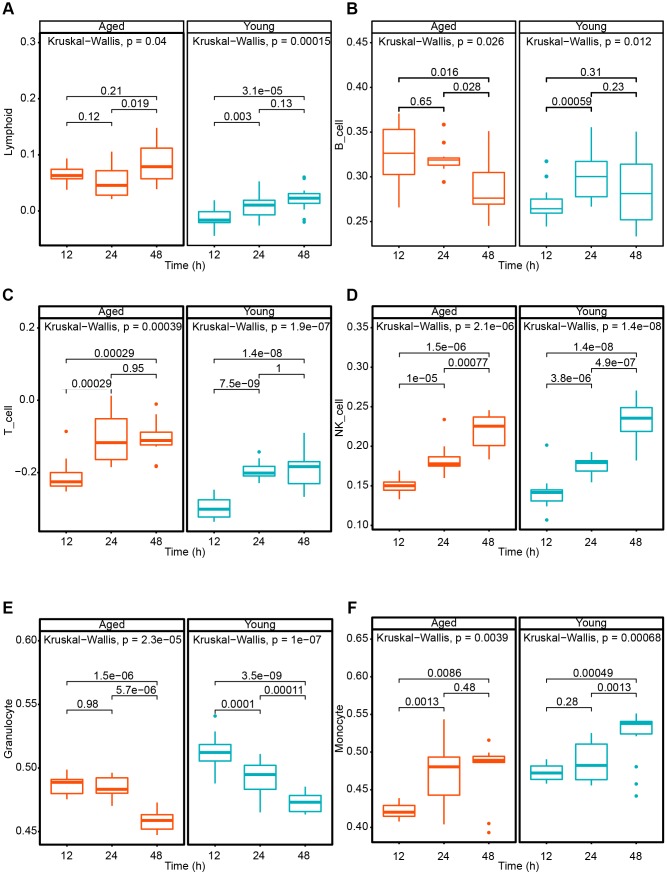
**The quantification of immune cell in SARS-CoV infected different age groups mice for 12 and 24 hours based on ssGSEA method.** (**A**) IL-1α; (**B**) IL-1β; (**C**) IL-6; (**D**) IL-7; (**E**) IL-10; (**F**) IL-23α.

To explore the immune differences between young and aged mice, we analyzed the cytokine variation after SARS-CoV infected for 12 and 24 hours. The results showed that several cytokines increase more significantly in aged mice than young mice (Figure 6). It indicated that coronavirus may cause more severe cytokine storms in elderly patients. To quantify the immune response on cell level, we applied ssGSEA method to compare the variation of different immune cells of aged and young mice after SARS-CoV infection. The level of T cells, NK cells and monocytes increased significantly both in aged and young mice. Lymphoid cells show an elevated level in young mice but remained stable comparatively in aged mice. And granulocytes tend to decrease both in aged and young mice after the infection. Interestingly, monocytes aged mice increased more quickly (24h) than in the young mice (48h) (Figure 7). The results showed that coronavirus infection can cause strong immune response in both young and old mice. Lymphocyte-mediated immune responses are more severe in young mice, but monocyte-mediated immune responses are more rapid in older mice.

### Clinical immunoassay of COVID-19 patients

**Figure 8:**
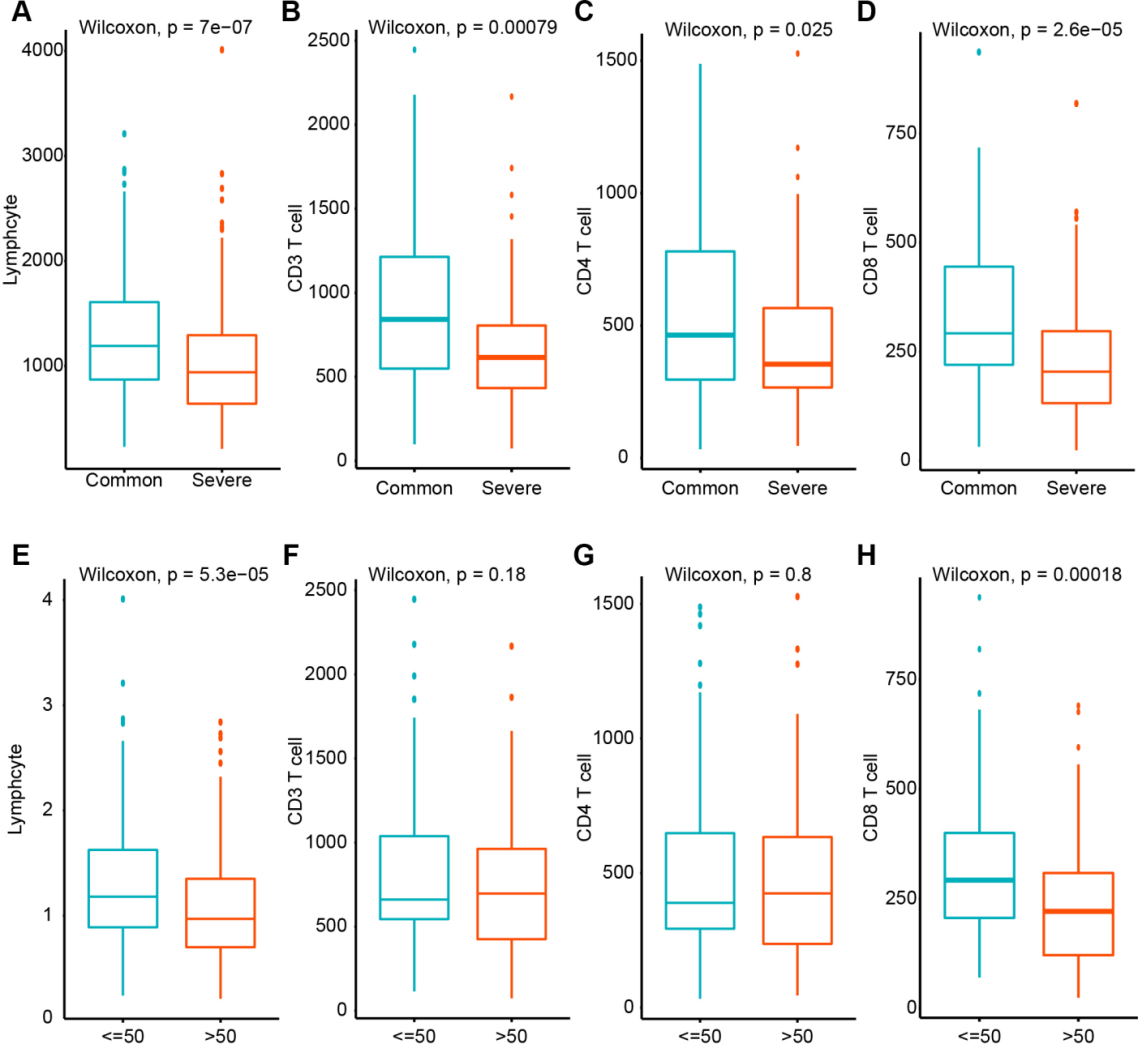
**The quantification of total lymphocytes, CD3+, CD4+ and CD8+ T lymphocytes from peripheral blood from COVID-19 patients by flow cytometry.** (**A**–**D**) Count variation between common and severe type disease. (**E**–**H**) Count variation between different age groups.

For further study, we analyzed immune cells in peripheral blood of 463 patients with COVID-19 disease (Table 1). We found that total lymphocytes, CD3+, CD4+ and CD8+ T lymphocytes significantly went down in the severe type patients compared to the common type (Figure 8) which indicated SARS-CoV-2 can impose hard blows on human lymphocyte resulting in lethal pneumonia. Moreover, total lymphocytes, and CD8+ T lymphocyte counts decreased more severely in patients >= 50 years old than those below 50 which suggest that young patients are more likely to bounce back. And CD3+ or CD4+ lymphocyte counts showed no significant difference between different age groups.

**Table 1:** Characteristics of 463 COVID-19 patients.

	**Overall (%)**	**Severe (%)**	**Common (%)**	***P* value**
Age (year)	51(43-60)	54(46-64)	49(42-58)	<0.001
15~49	208/463(44.92)	60/181(33.15)	148/282(51.06)	<0.001
50~64	177/463(38.23)	79/181(43.65)	98/282(32.62)	0.054
≥ 65	78/463(16.85)	42/181(23.20)	36/282(12.77)	0.034
Gender				
Male	244/463(52.70)	99/181(54.70)	145/282(51.42)	0.492
Female	219/463(47.30)	82/181(45.30)	137/282(48.58)	
Symptoms				
LOS (day)	12(9-14)	13(9-16)	11(9-14)	0.006
Fever (°C)	38.5(38-39)	38.9(38.2-39)	38.4(37.8-39)	<0.001
Cough	356/463(77.06)	145/181(80.11)	211/282(74.82)	0.188
Difficulty breathing	194/463(41.90)	82/181(45.30)	112/282(39.72)	0.234
Expectorant	146/463(31.53)	64/181(35.36)	82/282(29.08)	0.156
Fatigue	130/463(28.08)	49/181(27.07)	81/282(28.72)	0.699
Muscle ache	61/463(13.17)	21/181(11.60)	40/282(14.18)	0.423
headache	25/463(5.40)	10/181(5.52)	15/282(5.32)	0.924
Diarrhea	13/463(2.81)	4/181(2.21)	9/282(3.19)	0.533
Sore throat	13/463(2.81)	3/181(1.66)	10/282(3.55)	0.230
Runny	11/463(2.38)	3/181(1.66)	8/282(2.84)	0.618
Hemoptysis	9/463(1.94)	4/181(2.21)	5/282(1.77)	0.990

*LOS: Length of stay.

## DISCUSSION

Pathological manifestations of COVID-19 greatly resemble what has been seen in SARS and MERS infection which massive interstitial inflammatory infiltrates diffused in the lung [6]. The cellular fibromyxoid exudate which caused severe alveolar impairment from postmortem autopsy indicates the cytokine storm may play a critical role in patient rapid death. In this study, we found that genes coding interleukins(Il-1α, Il-1β, Il-6, Il-10), chemokines (Ccl2, Ccl3, Ccl5, Ccl10), and interferons (Ifn-α2, Ifn-β1 and Ifn2) raised significantly in SARS-CoV treated mice within 24h which in line with the elevated infiltration of T cells, NK cells and monocytes. And similar pattern of cytokine projection were found in the MERS-CoV infected group.

Investigating the inflammatory profile in SARS and MERS may advance our knowledge of the immune-pathological process in COVID-19 treatment. In this study, we reviewed SARS-infected mice and MERS-treated human micro vascular endothelial cells to clarify the association between temporal changes in cytokine/chemokine profiles and the six immune cell infiltration patterns. We retrospectively reviewed the clinical data of 463 cases with common and severe type COVID-19, who discharged before February 6, 2020. We found that severe type of patients suffered more serious symptoms like higher fever and took more time to recover which may suggest the fluctuation of immune indices is of predictive value.

To explore the specific mechanism of immune environment changes, we analyzed potential influencing factors. Cytokines, not merely aid in the process of antimicrobial immunity but are liable for immune-pathological damage to owner cells, causing significant morbidity or even fatality in multiple respiratory disorders as well [17, 18]. Chemokines like CXCL10 (IP10) and CCL2 (MCP-1) proved to be up-regulated in monocytes/macrophages by SARS-CoV which is consistent with our results [19]. The clinical progression of MERS cases proves that secretion of monocyte chemo-attractant protein-1 (MCP-1), CXCL10 is out of control [20]. Pro-inflammatory cytokines (IL-6, CCL5), and interferon-stimulated genes (CXCL10) are involved in Toll-like receptors (TLR) signaling [21]. These molecules are effectors on the process of respiratory virus infections towards the context of Acute Respiratory Distress Syndrome (ARDS) which is lethal to the COVID-19 patients [22]. IL-12 is the main cytokine secreted by DCs that manages the differentiation of CD4+ T cells into Th1 cells and serves essential duty in cell-mediated immunity. And IL-23 which includes in the IL-12 Family are predominantly pro-inflammatory cytokines which contribute critical roles in the growth of Th17 cells [23, 24]. Increased expression of IL-12 and IL-23 after SARS-infected lung tissue in mice may indicate the activated response of Th1 and Th17 cells which is observed in MERS victims as well [10]. Interesting, in the SARS-CoV infected cells, the ACE-2 was significantly correlated with neutrophils, NK cells, Th17 cells, Th2 cells, Th1 cells, DC which may call for further investigations [25].

IFN-α/β is regarded as one of the body’s primary antiviral defenses. IFN-β exerts its effects through intercellular communication resulting the induction of IFN-α/β and interferon-stimulated genes (ISGs), which make up an important aspect of host antiviral defense [26]. Notwithstanding, particular cell types, such as pDCs and monocytes, have been confirmed to produce more IFN than other cell types when viral infection committed [27]. And elevated level of IFN and monocyte infiltration in our analysis validates this. The innate immune response on the basis of pDCs and monocytes may play substantial role in the formation of the cytokine storm which damages the lung severely.

Lymphopenia is common in COVID-19 patients. Severe lymphocyte reduction occurred in about 10% of patients, especially in the heavy group, which is consistent with the latest reported results [28]. Flow cytometry showed that CD3+, CD4+ and CD8+ T lymphocytes had decreased to varying degrees. And aged patients suffered a more severe decrease in total lymphocytes and CD8+ T lymphocytes. About 40% of patients had a decrease in CD4 + T lymphocytes, and the incidence was higher in the heavy group than in the common group. This shows that SARS-CoV-2 may mainly attack lymphocytes in the body, which can cause the reduction of CD4 + T lymphocytes, resulting in decreased immune function and infection, and severe cases of severe pneumonia.

## CONCLUSIONS

In a word, we analyze the cytokine profiles in SARS-CoV infected mice and MERS-CoV infected human micro vascular cells. Interleukin (Il-1α, Il-1 β, Il-6, Il-10), chemokine (Ccl2, Ccl3, Ccl5, Ccl10), and interferon (Ifn-α2, Ifn-β1 and Ifn2) increased dramatically in SARS-CoV treated mice within 24h. As for MERS-CoV treated cells, interleukins (IL-6, IL-23α, IL-10, IL-7, IL-1α, IL-1β) and interferon (IFN-α2, IFN2, IFN-γ) showed a significant ascending trend in 24h. Subsequent analysis revealed elevated abundance of T cells, NK cells and monocytes in both young and aged mice group treated by SARS-CoV. And impaired lymphocyte system in severe and aged COVID-19 patients indicates the disease is more likely to progress when cytokines exhausted and functional lymphocytes suppressed. Thus, catching the window of treatment for COVID-19 according to these immune molecules may be critical.

## MATERIALS AND METHODS

### Microarray analysis

Microarray datasets related to gene expression were obtained from the GEO database For SARS-CoV dataset (GSE36969), young (8 weeks old) and aged (1 year old) female BALB/c mice were intranasally infected with 10^5 PFU of MA15 epsilon (SARS-CoV pathogenic virus). For MERS-CoV dataset (GSE79218), human microvascular endothelial cells were infected with MERSCOV002 (MERS-CoV pathogenic virus) or mocks and the 24h post-infection time point was picked for analysis. All gene expression datasets above were independently log2 transformed and quantile normalized in the linear models for microarray data (LIMMA) package in the R language environment.

### Clinical data

Patients who were diagnosed with COVID-19 and collected from Wuhan Jinyintan Hospital from January 1 to February 6, 2020 were collected. This study was approved by the Ethics Review Committee of Wuhan Jinyintan Hospital. Diagnostic criteria are according to the "Diagnosis and Treatment of New Coronavirus Pneumonia " issued by the General Office of the National Health and Health Commission as the diagnostic standard [13]. We classified patients into 2 types: (1) Common: fever, respiratory tract and other symptoms, with or without pneumonia manifestations on imaging; (2) Severe: meet any of the following: ① Respiratory distress, RR ≥ 30 beats / min; ② In resting state, refers to oxygen saturation ≤ 93%; ③ partial pressure of arterial oxygen (PaO2) / oxygen concentration (FiO2) ≤ 300mmHg.

### Statistical analysis

The Wilcoxon t-test were used to determine differences between two groups for continuous variables and the Kruskal – Wallis rank sum test for more than two groups, respectively. And we applied Single Cell Gene Set Enrichment Analysis (ssGSEA) to estimate the infiltration of immune cells [14] using the GSVA R package [15]. Fingerprint genes of granulocytes, monocytes, NK cells, activated and naive T cells, B cells and lymphoid cells are extracted from the previous study [16]. Statistical analyses were performed in the R (version 3.6.1) language environment and P-value <0.05 (two-sided) is considered to be significant.
